# Preliminary results of the TREBEA program, an outpatient comprehensive treatment for borderline personality disorder, in a region of northern Spain (Basque Country)

**DOI:** 10.1192/j.eurpsy.2026.11006

**Published:** 2026-07-31

**Authors:** P. Garcia De Pascual, F. Hernandez Alvarez, P. Leunda Echave, B. Gil Fernandez, R. Ule Pozo, Y. Rodriguez Garcia, F. Turriago Bernal, J. Alvarez Benito, C. Ludwig, E. Lopez de Munain Blanco

**Affiliations:** 1 Gipuzkoa Mental Health Network, Osakidetza; 2 Biogipuzkoa Health Research Institute, Donostia-San Sebastian, Spain

## Abstract

**Introduction:**

Personality disorders (PD) are a heterogeneous group of mental disorders characterized by maladaptive patterns of thinking, feeling, perceiving, and behaving. It is estimated that approximately 12% of the general population in Western countries meets the criteria for a PD diagnosis (Volkert *et al*., 2018).

Borderline Personality Disorder (BPD) is a PD characterized by a persistent pattern of instability in interpersonal relationships, self-image, emotions, and behavior. PDs have been associated with higher psychiatric comorbidity, 85% of people whith PD diagnosis have at least one other mental disorder of the DSM-5. They also have higher rates of self-harm, suicidal ideation, and suicide risk (Hiroeh *et al.*, 2001).

The TREBEA outpatient program is a comprehensive treatment for patients with BPD, which has an interdisciplinary and cross-cultural approach.This program takes place in Gipuzkoa, an area in northern Spain located in the Basque Country. Most interventions are based on dialectical behavioral therapy (DBT), most validated therapy and empirical evidence for the treatment of BPD (Gale *et al.*, 2022).

**Objectives:**

The TREBEA program would have three main objectives:

1. Clinical: Implementation of validated and effective therapeutic interventions for patients with BPD.
2. Research: Evaluation of the effectiveness of interventions implemented in our population and study of factors that influence the clinical course of patients with BPD.
3. Academic: Conducting training activities for the Gipuzkoa mental health network and training therapists and trainees

**Methods:**

Patients are referred from 13 mental health centers in Gipuzkoa, part of the Basque country (nothern Spain).

The assessment includes an extensive questionnaire on sociodemographic data, which provides information on medical history, clinical course, current symptoms, comorbidities, pharmacological treatment, psychosocial functioning, and other significant factors.

In adition, we also include a structured interview, The Diagnostic Interview for Borderlines-Revised (DIB-R) and various self-report questionnaires are administered, such as CORE-OM, BSL-23, and ICG-TLP.

This assessment is conducted before and after the intervention. The program lasts 16 weeks and is based on Dialectical Behavior Therapy (DBT). Patients can complete up to 32 weeks.

**Results:**

Patients included in the TREBEA program show a significant reduction in the frequency of self-harming and impulsive behaviors, a decrease in symptom severity, and an improvement in psychosocial functioning (Image 2). In addition, there is a reduction in the number of emergency room visits and hospital admissions at the end of the program and six months after the intervention.

**Image 1:**
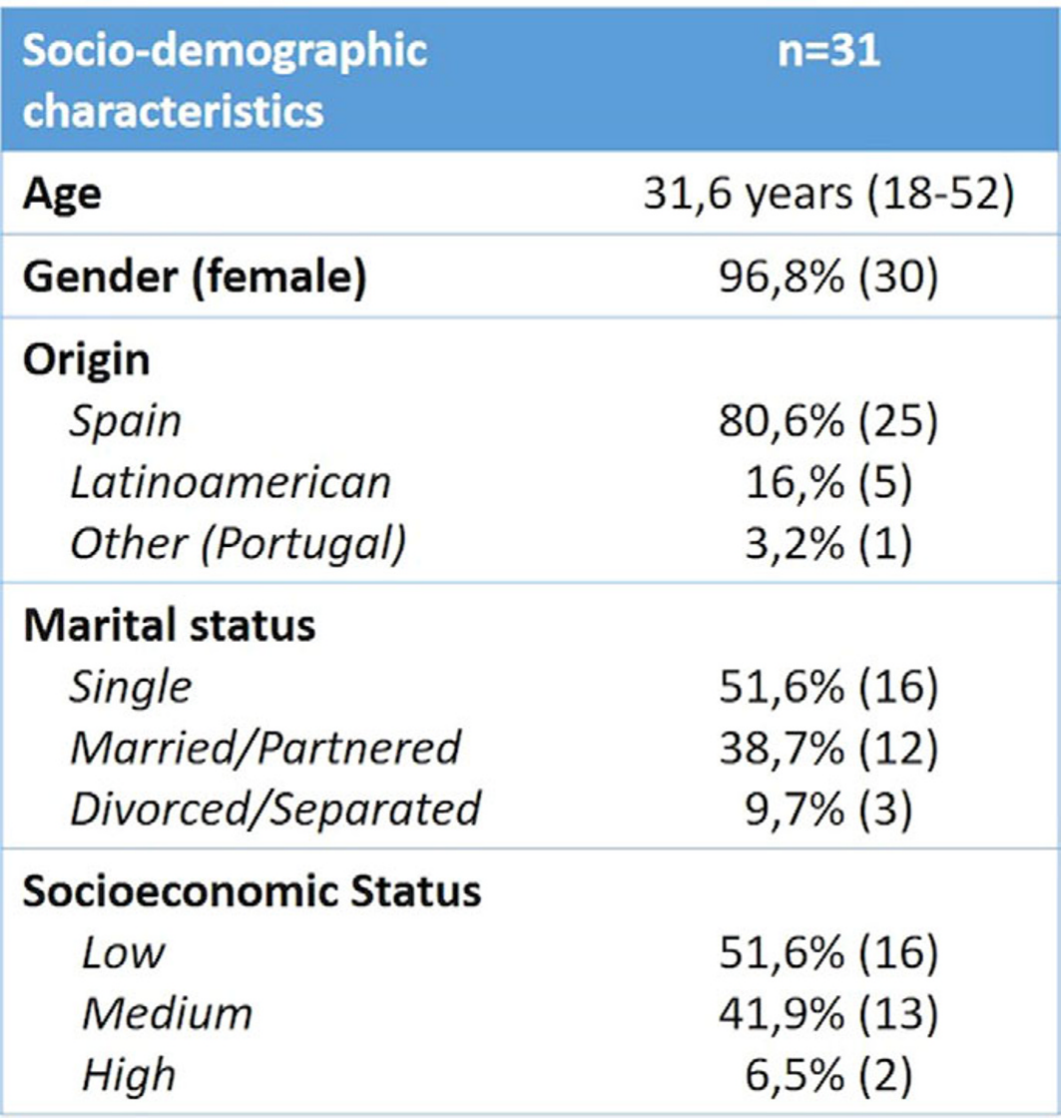
Image 1: Long description.

**Image 2:**
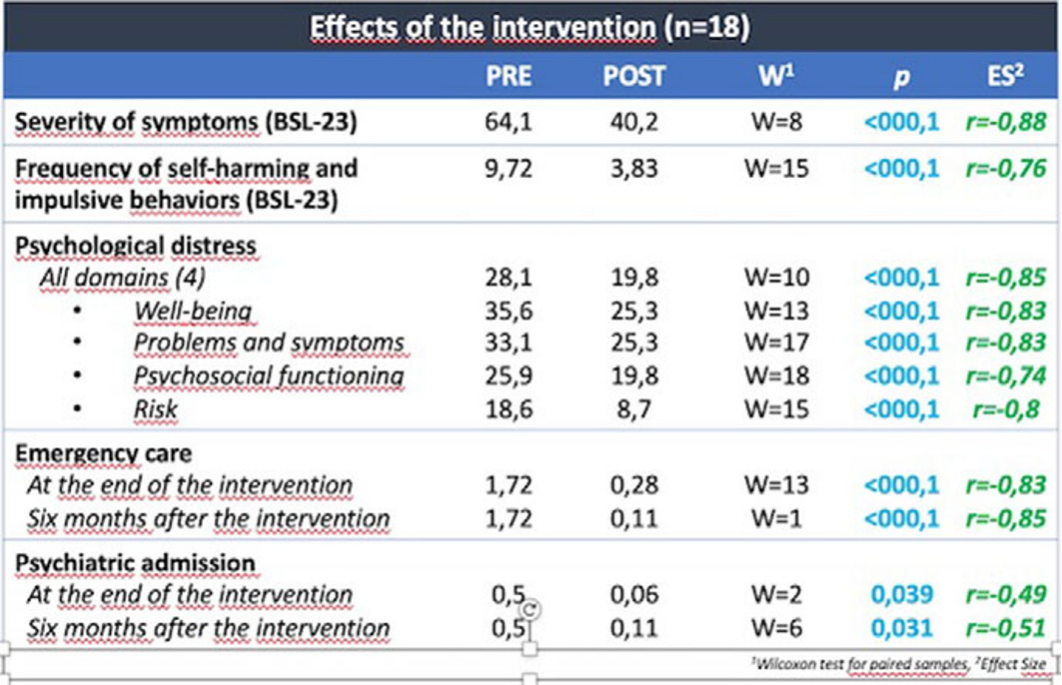
Image 2: Long description.

**Conclusions:**

The TREBEA program has been successfully implemented and has proven effective in reducing symptom severity and increasing patient functionality.

**Disclosure of Interest:**

None Declared

## Long descriptions

### Image 1: Long description

The table header is blue with white text stating Socio-demographic characteristics and n equals 31. The data rows are as follows:

* Age: 31,6 years (18-52).

* Gender (female): 96,8% (30).

* Origin:

- Spain: 80,6% (25).

- Latinoamerican: 16% (5).

- Other (Portugal): 3,2% (1).

* Marital status:

- Single: 51,6% (16).

- Married/Partnered: 38,7% (12).

- Divorced/Separated: 9,7% (3).

* Socioeconomic Status:

- Low: 51,6% (16).

- Medium: 41,9% (13).

- High: 6,5% (2).

Navigate back to Image 1:.

### Image 2: Long description

The table contains five columns: Outcome Measure, P R E, P O S T, W super 1, p, and E S super 2. All p-values are highlighted in blue and E S values in green.

* Severity of symptoms B S L-23: P R E 64,1; P O S T 40,2; W=8; p <000,1; r=-0,88.

* Frequency of self-harming and impulsive behaviors B S L-23: P R E 9,72; P O S T 3,83; W=15; p <000,1; r=-0,76.

* Psychological distress All domains 4: P R E 28,1; P O S T 19,8; W=10; p <000,1; r=-0,85.

* Well-being: P R E 35,6; P O S T 25,3; W=13; p <000,1; r=-0,83.

* Problems and symptoms: P R E 33,1; P O S T 25,3; W=17; p <000,1; r=-0,83.

* Psychosocial functioning: P R E 25,9; P O S T 19,8; W=18; p <000,1; r=-0,74.

* Risk: P R E 18,6; P O S T 8,7; W=15; p <000,1; r=-0,8.

* Emergency care at end of intervention: P R E 1,72; P O S T 0,28; W=13; p <000,1; r=-0,83.

* Emergency care six months after: P R E 1,72; P O S T 0,11; W=1; p <000,1; r=-0,85.

* Psychiatric admission at end of intervention: P R E 0,5; P O S T 0,06; W=2; p 0,039; r=-0,49.

* Psychiatric admission six months after: P R E 0,5; P O S T 0,11; W=6; p 0,031; r=-0,51.

Footnotes indicate 1 Wilcoxon test for paired samples and 2 Effect Size.

Navigate back to Image 2:.

